# Identification of Elongated Primary Cilia with Impaired Mechanotransduction in Idiopathic Scoliosis Patients

**DOI:** 10.1038/srep44260

**Published:** 2017-03-14

**Authors:** Niaz Oliazadeh, Kristen F. Gorman, Robert Eveleigh, Guillaume Bourque, Alain Moreau

**Affiliations:** 1 Viscogliosi Laboratory in Molecular Genetics of Musculoskeletal Diseases, Sainte-Justine University Hospital Research Center, Montreal, Quebec, H3T 1C5, Canada; 2 Department of Biochemistry and Molecular Medicine, Faculty of Medicine, Université de Montreal, Montreal, Quebec, H3T 1J4, Canada; 3 Department of Biological Sciences, California State University, Chico, CA 95929, USA; 4 Genome Quebec Innovation Center, McGill University, Montréal, Quebec, H3A 0G1, Canada; 5 McGill University, Montréal, Quebec, H3A 1A4, Canada; 6 Department of Stomatology, Faculty of Dentistry, Université de Montréal, Montreal, Quebec, H3T 1J4, Canada

## Abstract

The primary cilium is an outward projecting antenna-like organelle with an important role in bone mechanotransduction. The capacity to sense mechanical stimuli can affect important cellular and molecular aspects of bone tissue. Idiopathic scoliosis (IS) is a complex pediatric disease of unknown cause, defined by abnormal spinal curvatures. We demonstrate significant elongation of primary cilia in IS patient bone cells. In response to mechanical stimulation, these IS cells differentially express osteogenic factors, mechanosensitive genes, and signaling genes. Considering that numerous ciliary genes are associated with a scoliosis phenotype, among ciliopathies and knockout animal models, we expected IS patients to have an accumulation of rare variants in ciliary genes. Instead, our SKAT-O analysis of whole exomes showed an enrichment among IS patients for rare variants in genes with a role in cellular mechanotransduction. Our data indicates defective cilia in IS bone cells, which may be linked to heterogeneous gene variants pertaining to cellular mechanotransduction.

Idiopathic scoliosis (IS) is a complex pediatric syndrome that manifests primarily as an abnormal three-dimensional curvature of the spine. Eighty percent of all spinal curvatures are idiopathic, (MIM 181800) making IS the most prevalent form of spinal deformity. With a global incidence of 0.15% to 10% (depending on curve severity)^1^, IS contributes significantly to the burden of musculoskeletal diseases on healthcare (http://www.boneandjointburden.org). Children with IS are born with a normal spine, and the abnormal curvature may begin at different points during growth, though adolescent onset is the most prevalent^2^. Idiopathic scoliosis is diagnosed by ruling out congenital defects and other causes of abnormal curvature, such as muscular dystrophies, tumors, or other syndromes.

The etiology of idiopathic scoliosis is unknown largely because of phenotypic and genetic heterogeneity. Curve magnitude is highly variable and the risk for severe curvature is not understood beyond the observed female bias. Although a genetic basis is accepted, genetic heterogeneity has been implicated in several familial studies^3^,^4^, and numerous genome-wide association studies (GWAS) have detected different loci with small effects^5^. Despite such genetic correlations, no clear biological mechanism for IS has emerged. It is likely that IS phenotypic heterogeneity is a consequence of genetic variations combined with biomechanical factors that are influenced by individual behavioral patterns. As a musculoskeletal syndrome, biomechanics are thought to have an important role in the IS deformity. Pathological stressors applied to a normal spine or normal forces on an already deformed spine have been studied for a role in curve predisposition and progression^6^. For example, factors that contribute to spinal flexibility, sagittal balance, shear loading on the spine, and compressive or tension forces may contribute to the ‘column buckling’ phenotype associated with IS^7^,^8^,^9^. Furthermore, therapeutic options available for IS, bracing and corrective surgery, approach the disease from a mechanical perspective, and successful outcomes depend on understanding the complex biomechanics of the spine. In this paper, we report evidence supporting an association between IS and mechanotransduction through the non-motile microtubule-based signaling organelle known as cilium.

Primary cilia are antenna-like organelles that transmit chemical and mechanical signals from the pericellular environment^10^,^11^. They are found in the cells of all human tissues (except blood), including bone, cartilage, tendons, and skeletal muscles (a comprehensive list of tissue types and cell lines with primary cilia can be found at: http://www.bowserlab.org/primarycilia/ciliumpage2.htm). In addition to functions linked to olfaction, photo and chemical sensation, recent studies have established a mechanosensory role for primary cilia in tissues, such as the kidney, liver, embryonic node, and bone structure (the mechanosensory role of cilia in bone is reviewed by Nguyen *et al*.)^12^. As the most recent established role for cilia, mechanisms for mechanosensation are not yet entirely understood. For example, the involvement of calcium channels, in response to cilia bending following a fluid movement, is yet a matter of debate and might vary depending on the examined tissue^13^,^14^.

As a mechanosensor in bone, the primary cilium can transduce fluid flow induced shear stress occurring within the canaliculi that interconnect osteocytes as well as strain-related mechanical stimuli in pre-osteoblasts^15^. The load-induced fluid flow in bone canaliculi is recognized to play a role in maintaining bone homeostasis through bone resorption and formation cycles (i.e. bone tissue remodeling)^16^. Cilia mediate the transduction of this fluid flow to mesenchymal stem cells (MSCs), and is implicated in osteogenic gene expression and lineage commitment^17^. Mechanical loading modulates the incidence and length of primary cilia in cells, such as chondrocytes, in which cilia direction affects the direction of growth in growth plates^18^. Mechanical loading has also been shown to induce bone cell proliferation through a cilia-dependent mechanism^15^. Interestingly, skeletal disorders are a common feature in several human ciliopathies, such as Jeune syndrome and short rib-polydactyly^19^.

Considering the role of primary cilia in mechanotransduction, our hypothesis was that idiopathic scoliosis is a ciliopathy-like condition and that the genetic architecture of IS may involve enrichment of pathogenic variants in ciliary genes. To explore this idea, we reviewed established ciliary genes for an association with spinal curvature and examined the function of validated IS genes for connections to cilia. We found that numerous ciliary genes induce a spinal curvature phenotype when knocked down in animal models, and that scoliosis is associated with many human ciliopathy syndromes^20^,^21^. Additionally, the majority of confirmed IS associated genes are connected to cilia structure or function (Table 1). To test our hypothesis, we examined bone-derived cells and exomes of patients and controls.

Confocal images of primary osteoblast cultures, derived from bone fragments obtained intraoperatively at the time of the spine surgery, revealed that IS patients have longer primary cilia and an increased density of cells with elongated cilia. To investigate the functional consequences of the IS cilia phenotype, we applied controlled mechanical strain to bone cell cultures and examined the expression of osteogenic, mechanosensitive, and signaling genes at several time points. To explore a direct genetic correlation between cilia and IS in our French-Canadian cohort, we used SNP-set Kernel Association Test – Optimal Unified approach, or SKAT-O^22^ to analyze exomes of 73 IS patients and 70 controls. The results suggest that IS may be caused by variants in genes related to cellular mechanotransduction, but is not restricted to ciliary genes.

## Results

### Osteoblasts of IS patients have longer cilia

**Figure 1:**
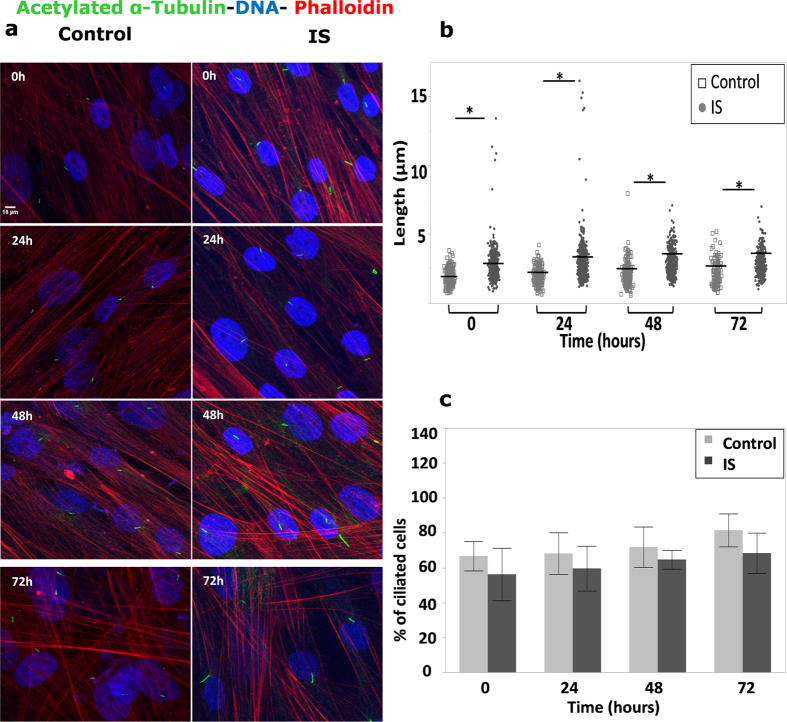
Morphology of primary cilia in osteoblasts from IS and controls. (**a**) Immunofluorescence micrographs of IS and control osteoblasts at 0, 24, 48, and 72 hours following serum-starvation. Cells were stained for acetylated α-Tubulin (green), F-Actin (red), and Hoescht (blue). Long cilia (green) are visible in IS patients, at all time-points. (**b**) Elongated primary cilia appear more frequently in IS bone cells (4 IS vs. 4 controls assayed in duplicate, from 5 × 5 stitched tile images (50 fields) per sample). (**c**) Percentage of ciliated cells is not significantly different between IS and control cells (n ≤ 1,000 count per individual). Error bars are constructed using 1 standard error from the mean. Statistical analysis was performed with t-test using JMP-11, *P < 0.005.

To assess whether there is an observable defect associated with primary cilia in IS patients, we chose to look at osteoblast cells derived from bone specimens obtained during surgery. All samples were from age matched adolescent female subjects. FBS deprivation was used to promote ciliogenesis and differentiation. We examined cilia morphology using anti-acetylated α-tubulin immunofluorescence staining prior to and after 24, 48, and 72 hours (h) starvation in primary osteoblasts from 4 IS patients and 4 non-scoliotic trauma patients used as controls (Fig. 1a). The fraction of ciliated cells and cilia length were quantified in fixed and stained cells. Measurements were acquired from 5 × 5 stitched tile images per sample, in duplicate (50 fields). We found that the cilia in IS-derived cells were approximately 30% to 40% longer than cilia in control cells (Fig. 1b and Table 2). We also observed that IS cells showed a reduced incidence of ciliated cells compared to controls, although the difference did not reach the statistical significance (Fig. 1c). To validate the staining of cilia, double immunostaining was performed on fixed IS osteoblasts using anti-Ninein, as the basal body marker or anti-IFT88 to stain the length of cilia alongside the anti-acetylated α-Tubulin (Supplementary Fig. S1).

### IS and control osteoblasts grow at the same rate

**Figure 2:**
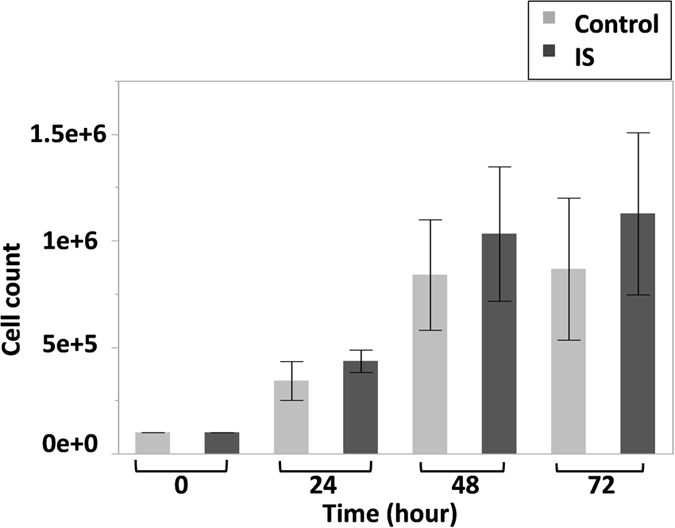
Similar growth rates among IS and control cells. There is no significant difference in the average cell number between control and IS patient cells at any given time point (n = 8: 4 IS vs. 4 controls). Plates were seeded with 100,000 cells per well, in triplicate, for each patient and control. Each error bar is constructed using 1 standard error from the mean.

Cilia assembly, disassembly, and length have been associated with cell cycle and control of cell proliferation^23^. To investigate if there is a correlation between longer cilia in IS patients and a differential growth rate, we assayed proliferation by counting viable cell (Trypan Blue stained) number as a function of time. Cell proliferation rate was various in all the samples as evidenced by the error bars (Fig. 2). It seems that IS cells increase in number slightly faster than controls but the difference does not pass the significant threshold at any of the three time points analyzed (24, 48 and 72 h).

#### Lithium Chloride (LiCl) increases the length of primary human osteoblasts

Lithium chloride has previously been used to induce longer cilia in some cell types^24^. To examine whether the LiCl induced elongated cilia is similar to the long cilia phenotype observed in IS cells, we treated the control cells with three different concentrations of LiCl (1 mM, 10 mM and 50 mM)^24^ and examined their cilia with confocal microscopy. Primary human osteoblasts died after 24 h of exposure to 50 mM LiCl. However, we measured a significant increase in the cilia length at both 1 mM (3.94 ± 1.24 μm) and 10 mM (5.81 ± 1.72 μm) LiCl treated cells compared to non-treated cells (2.18 ± 0.48 μm). LiCl treatment did not affect the percentage of ciliated cells (Supplementary Fig. S2).

### IS cells have impaired biomechanical response

We evaluated the functional response of IS cells having long cilia by monitoring changes in expression for several mechanoresponsive genes under fluid flow, at three time points (4 h, 8 h, 16 h). We applied a 1 [Pa] shear stress (the magnitude at the center of the dish) in 1 [Hz] frequency, which corresponds to a Womersley number of 8, equations (1, 2 and 3). The biomechanical parameters were chosen to be physiologically relevant based on the reported frequency spectra of forces affecting the human hip during walking, (1–3 Hz)^25^, and the Womersley number estimated for cerebrospinal fluid motion in the spinal cavity (5–18)^26^.

**Figure 3:**
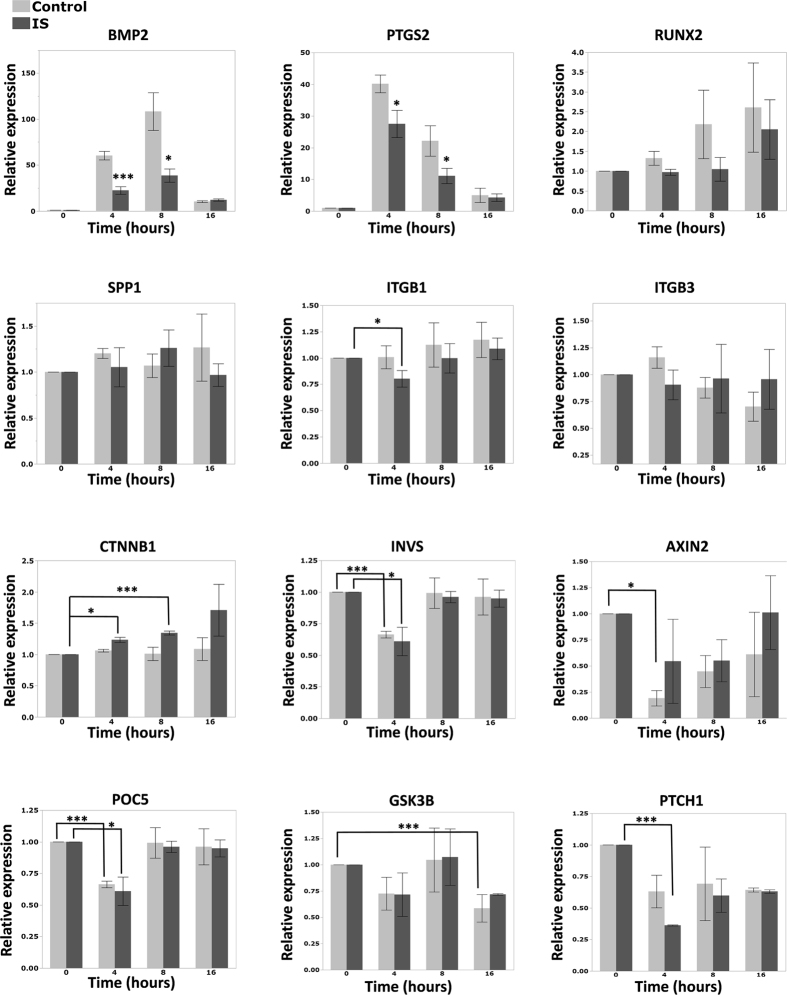
Biomechanical response profile of human primary osteoblast from IS patients with elongated cilia vs. control. We used RT-qPCR to examine the effect of oscillatory fluid flow on gene expression after 4, 8, and 16 hours of stimulation. Gene expression in every sample has been normalized to two endogenous controls (GAPDH and HPRT). The 0 h of every sample has been defined as its calibrator. The graphs represent the fold changes at each time point, compared to the calibrator. For each gene, the two groups (control and IS) were compared at each time point using a pairwise t-test. In addition, the expression at 0 h for IS was compared to each of the other time points (4 h, 8 h and 16 h) using separate pairwise t-tests. For a post hoc Bonferroni analysis, the maximum number of comparisons per gene is 6, three comparisons per question (i.e. three comparisons per family of test). Even if we consider each gene as a family (i.e. six comparisons), using this formula (FWER = 1 − (1 − α)M^88^) the family-wise error rate (FWER) would be: 1−(1–0.05) 6 ≈ 1 − 0.73 ≈ 0, 26. Solving the Bonferroni (0.26/6) new α would be 0.043 which does not affect our findings. For example CTNNB1 results at 4 (p = 0.03) and 8 hours (p = 0.008) will still be significant. It is the same case for POC5 4 h (p = 0.01) but ITGB1 with a p = 0.047 will not pass the test. Overall, the multiple test error is not significant in our analysis and it will not change the results. Genes were chosen based on the following characteristics: Biomechanically responsive genes in bone tissue: BMP2, PTGS2 (COX2), RUNX2, SPP1 (OPN); role in mechanotransduction through cilia: ITGB1, ITGB3; indicator of Wnt pathway activity: CTNNB1; GSK3B, AXIN2, INVS; indicator of Hedgehog signaling: PTCH1; or implicated in an IS study: POC5. Each error bar is constructed using 1 standard error from the mean. n = 8, (4 IS vs. 4 controls) for all genes except CTNNB1, where n = 4 (2 IS vs. 2 controls).

Differential gene expression was compared for IS vs. controls at each time point, and then the whole response profile of each gene was examined. For each gene, after normalization to two endogenous controls (GAPDH and HPRT), the baseline expression level at 0 h (before treatment) of every sample was defined as its own calibrator. The gene expression for all time points for each sample was compared to its own 0 h (which has a RQ value of 1). The results are shown as fold changes compared to the calibrator (Fig. 3). We asked one question per gene: is there any difference between IS and control at each time point? For three genes (ITGB1, CTNNB1 and POC5) that showed a different overall expression pattern in IS vs. controls, we asked a second question: is there a significant difference in gene expression before and after flow? Each gene has been analysed independently using a pair wise t-test for each question followed by a post-hoc Bonferroni correction. Concordant with previous findings regarding biomechanical induction and the expression of osteogenic factors^12^,^17^,^27^, our assay showed a dramatic increase in Bone morphogenetic protein 2 (BMP2) and Cyclooxygenase-2 (COX2) expression in IS and controls, following 4 and 8 hours of fluid flow induction. However, for both genes, the IS response was significantly less than controls (Fig. 3). The response for Runt-Related Transcription Factor 2 (RUNX2) in IS patients, while not significant, is also less than what we observed in controls. We also tested the expression of Secreted Phosphoprotein 1 (SPP1, also known as Osteopontin or OPN) as an osteogenic factor in bone, and did not observe a biomechanical response in IS or control cells (Fig. 3). The modified responses to mechanical stress observed in this study corroborate those previously reported in human mesenchymal stem cells (MSCs)^17^. Expression of integrin beta 1 (ITGB1) and integrin beta 3 (ITGB3) were monitored due to their possible role in transmitting mechanical signals in bone^28^. The expression of ITGB1 did not notably change during 16 h of flow application in controls, while a significant decrease in expression was observed in IS cells (p = 0.025) at 4 hours post flow. ITGB3 expression did not significantly change in IS or control cells. Cilia are well known for their regulatory effect on major signaling pathways notably Wnt and Hedgehog^19^,^29^. Beta-catenin, a main player in the canonical Wnt pathway^29^,^30^,^31^, is localized to the cilium^32^. We found the expression of beta-catenin (CTNNB1) did not change in control osteoblasts as it has been shown previously^31^, while IS cells showed a significant continual rise in CTNNB1 expression in response to flow application (p at 4 h = 0.03, 8 h = 0.008). To further explore the role of Wnt signaling in IS, we also tested the expression of Axin2, GSK3B (Glycogen Synthase Kinase 3 Beta), and INVS (Inversin), after exposure to flow. Although pairwise comparisons between IS and controls did not show a significant difference in Axin2 expression, IS cells showed a reduced response to mechanical stimuli. This was most apparent after 4 hours of flow application, where control cells had a significant reduction in expression but IS cells did not. Inversin and GSK3B responses were not significantly different between IS and controls. To examine the possibility of Hedgehog pathway involvement in the IS mechanical response, we measured the activity of PTCH1 (patched) and Gli1 as established indicators of HH pathway activity^24^. PTCH1 expression decreased among both IS and controls at all times, although after 4 hours we observed a more significant response among IS patients (Supplementary Fig. S3). GLI1 expression was not detected after 35 cycles in two attempts of RT-qPCR (data not shown).

Fuzzy planar cell polarity (FUZ), Protein Of Centriole 5 (POC5), and Ladybird homeobox 1 (LBX1) genes were added to the experiment following our exome analysis, and recent published scoliosis genetic studies^3^,^33^,^34^. For FUZ, response profiles were not different among IS and controls (data not shown). Although, we did not see any significant differential expression between IS and controls for POC5, it is worth mentioning that its expression decreased almost by half at the 4 hour point in both IS and controls (p < 0.05), suggesting a role in early stages of mechanotransduction response (Fig. 3). LBX1 expression was not detected after 35 cycles in two attempts of RT-qPCR (data not shown).

#### LiCl treated control cells also show impaired biomechanical response

**Figure 4:**
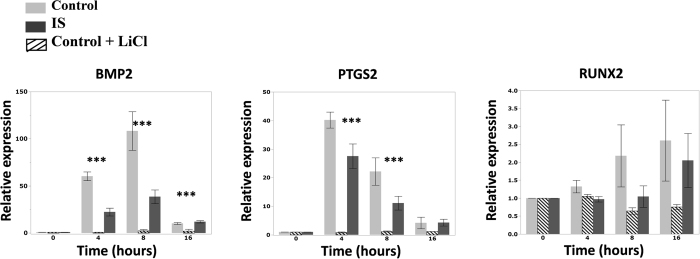
Osteogenic factors response profile of primary human osteoblasts from control, IS and LiCl treated controls under mechanical stimulation. We used RT-qPCR to examine the effect of oscillatory fluid flow on gene expression after 4, 8, and 16 hours of stimulation. Gene expression in every sample has been normalized to two endogenous controls (GAPDH and HPRT). The 0 h of every sample has been defined as its calibrator. The graphs represent the fold changes at each time point, compared to the calibrator. Three groups were compared using Anova and followed by post-hoc Tukey test to analyze the significant results. 10 mM LiCl treatment for 24 h significantly suppresses the expression of tested osteogenic factors under mechanical stimulation compared to non treated controls (p < 0.0001). The response pattern of LiCl-Control group follows what is observed in IS but is dramatically exaggerated. It is of note that response is not completely lost Statistical analysis was performed with Anova, followed by Tukey test using JMP-12.

We examined the mechanosensory response of control osteoblasts treated with LiCl for comparison to the non-treated control and IS patient cells. An ANOVA was used to compare gene expression levels among the three groups and a Tukey test was used for post-hoc analysis of significant results. In LiCl treated control cells the three osteogenic genes (BMP2, PTGS2, RUNX2) showed a dramatic decrease in expression in response to flow that was significant for BMP2 and PTGS2. This response had a similar but exaggerated pattern compared to that observed in IS cells (Fig. 4). Of the other genes tested, none showed a significant differential response (Supplementary Fig. S3).

#### LiCl affects the expression of Wnt signaling indicators and osteogenic factors

To investigate the possibility of inducing an IS-like phenotype in control cells using LiCL, we tested the expression of several osteogenic and signaling factors in IS cells and LiCl-treated controls in comparison with non-treated controls (Table 3). Primary human osteoblast cells from the control group were treated with 10 mM LiCl, for 24 h (Based on work by Thompson *et al*.)^24^. As expected^24^, LiCl caused a decrease in GSK3B expression. Inhibition of GSK3B is associated with the activation of canonical Wnt signaling, and in both LiCl treated cells and IS cells there was an increase in beta-catenin expression (3.4-fold and 5.8-fold respectively). However, IS patients did not have altered basal expression for GSK3B, instead they showed a 2-fold decrease in AXIN2 expression, which was not the case in LiCl-treated control cells. Both IS and LiCl treated cells had significantly decreased levels of RUNX2. LiCl treated cells had a 3.9-fold increase in PTGS2 (COX2) expression that was not observed among IS cells. Idiopathic scoliosis cells had a 2-fold overexpression of SPP1 compared to controls but treated cells showed a 2.7-fold decrease in expression.

### Whole Exome Sequencing (WES) results

We used whole exome sequencing to test the hypothesis that rare variants in ciliary genes might be causal for IS. We performed exome sequencing on peripheral blood DNA sampled from 73 IS and 70 matched controls using the Agilent SureSelect Human All Exon 50 Mb v3 capture kit and the Life Technologies 5500 SOLiD Sequencing System. Variants were called and annotated using a customized bioinformatics pipeline including SAMTOOLS, GATK and Picard program suites^35^. To reduce the number of likely variants, we subsequently filtered the total variant set to remove those with a minor allele frequency greater than 5%, as well as variants not in or adjacent to protein-coding exons. After filtering, our dataset included 73 IS patients, 70 controls, 8544 genes, and 16,384 variants. We used SKAT-O to survey our exome data under two different weighting parameters: in favor of lower frequency variants (Madsen Browning weighting, Set I), and in favor of variants with projected deleterious effects and pathogenicity (Combined Annotation Dependent Depletion: CADD weighting, Set II). Since the underlying biology of idiopathic scoliosis is not understood, an omnibus test such as SKAT-O is considered more powerful than a burden test because it does not make assumptions regarding direction or size of variant effect^36^. Analysis using Madsen Browning weighting (Set I) identified 259 genes and analysis using CADD weighting (Set II) identified 240 genes that are significant (p ≤ 0.01) after correction for multiple testing. The Sets were compared and genes that were significant in both (n = 120; Supplementary Table S1) were considered candidates for idiopathic scoliosis. This list was examined for ciliary genes using the SYSCILIA gold standard list^37^ and the Kim *et al*.^38^ list as references, along with inquiries using Google search engine. Fuzzy planar cell polarity protein (FUZ) is the only known ciliary gene in both data lists. However, there is a greater number of variants in controls compared with cases (12 controls with at least one variant vs 1 patient). Of the candidate genes, the 25 most significant (p < 0.001) were further examined to determine the number of patients and controls having at least one variant. Seven of these genes have greater variant enrichment among patients: CD1B, CLASP1, SUGT1, HNRNPD, LYN, ATP5B, AL159977.1 (Supplementary Table S1).

We also looked at the variant profile for each of the four IS patients used in our cellular analyses, to see if there are shared genes with variant enrichment. Controls could not be examined because the cohort used for molecular work differs from the genotyped control cohort. Control bone tissue was obtained intraoperatively from non-scoliotic trauma patients whereas the genotyped controls were from a non-surgical cohort. None of the genes identified in our combined SKAT-O table were shared among all the four tested patients, but they all have variants in either CD1B, CLASP1, or SUGT1. The CDK11A gene is represented among three patients, but in the exome cohort, nearly all patients and controls have at least one variant for this gene (Supplementary Fig. S4).

## Discussion

In the current study, we provide experimental evidence that correlates idiopathic scoliosis (IS) with morphological and functional abnormalities in cilia. Elongated primary cilia in IS patient osteoblast cells is a novel observation that may be linked to the etiology of the disease. We examined the possibility of inducing IS like phenotype in control osteoblasts by increasing their cilia length with LiCl treatment. Using SKAT-O to survey IS and control exomes, we suggest that idiopathic scoliosis is a result of heterogeneous defects in genes pertaining to the cellular mechanotransduction machinery. Currently, idiopathic scoliosis is exclusively defined by its clinical phenotype and genetic studies have failed to identify a consensus ontology. Therefore, this study introduces an important biological context to understand mechanisms associated with the disease.

We found that cilia in IS cells are significantly longer across all measured time points, but the most conspicuous length differences are visible before starvation and at 24 hours after starvation (Fig. 1b), suggesting an abnormality in cilia formation at early stages of cilia growth. Although we did not see any significant differences in the percentage of ciliated cells before starvation, long cilia (up to 13 μm in few cells) were visible in pre-starved IS bone cells and not controls. This could suggest actin organization impairment, considering that actin polymerization inhibitors induce longer cilia and facilitate ciliogenesis independently of starvation^38^. Irregularity in the control of cilia length has been associated with cytoskeletal disruption and actin dynamics, either due to genetic mutation^38^ or in response to mechanical stress^39^. While there is no statistically significant difference in the incidence of cilia between controls and IS, there does seem to be a trend of IS cells having a lower incidence than control at every time point. This might be an indicator of cell cycle irregularities, considering the tight correlation of cilia differentiation to cell cycle progression. Our proliferation assay confirmed that the IS cilia phenotype is independent of cell proliferation, but we did not rule out the existence of any impairment in different phases of cell cycle. In an attempt to induce an IS like phenotype we treated control osteoblasts with LiCl, which significantly increased the length of their cilia. The primary cilium participates in cell signaling, cell cycle regulation and mechanotransduction during development, homeostasis, and regeneration. The length of the primary cilium has an important effect on the quantity of forces transmitted to internal cell components, with longer cilia inducing higher strains compared with shorter ones^11^. In addition, the higher deflection of a long cilium will affect the regulation of certain molecular responses, such as stretch-sensitive channels, and transporter proteins, plus key pathways such as Hedgehog, Wnt, and platelet-derived growth factor signaling^11^,^28^,^40^,^41^,^42^,^43^.

Cilia have been shown to play important roles in both the canonical Wnt/beta-catenin and non-canonical Wnt/planar cell polarity pathways^29^, although these roles are not well understood. The Wnt/beta-catenin pathway controls gene expression through the stability of beta-catenin^32^. Cilia are known to restrain canonical Wnt signaling^32^ by beta-catenin regulation^44^. The absence of cilia alters beta-catenin activity and increases Wnt signaling, while multiciliated cells show an inhibited Wnt response^44^. The effect of elongated cilia on Wnt activity is not fully understood. In both IS cells with naturally longer cilia and LiCl treated cells with induced elongated cilia we observed an elevation in basal levels of CTNNB1 (beta-catenin) gene expression. LiCl treatment is known to mimic canonical Wnt activation through the inhibition of GSK3B, and subsequent prevention of beta-catenin protein degradation. Although we did not observe altered expression of GSK3B among IS cells, there was a decrease in basal expression of AXIN2, another negative regulator of canonical Wnt signaling. Furthermore, in both induced and IS elongated cilia phenotypes there was a decrease in basal levels of RUNX2 expression. This is consistent with LiCl induced inactivation of GSK3B, and the fact that GSK3B and Wnt are among RUNX2 regulators^45^. We assayed Inversin (INVS) as an indicator of non-canonical Wnt activity while PTCH1 and GLI1 were chosen as players in Hedgehog signaling, based on the previous works by Simons^46^ and Thompson^24^, respectively. We did not observe significant basal expression differences among IS, LiCl-treated, and controls for INVS or PTCH1. GLI1 showed a slight decrease (0.6-fold) in IS cells compared to controls but its expression was not detected in LiCl treated cells. In summary, the basal gene expression profile of the IS cells with elongated cilia has similarities to the LiCl induced elongated cilia in control cells, suggesting altered canonical Wnt signaling (Table 3).

**Figure 5:**
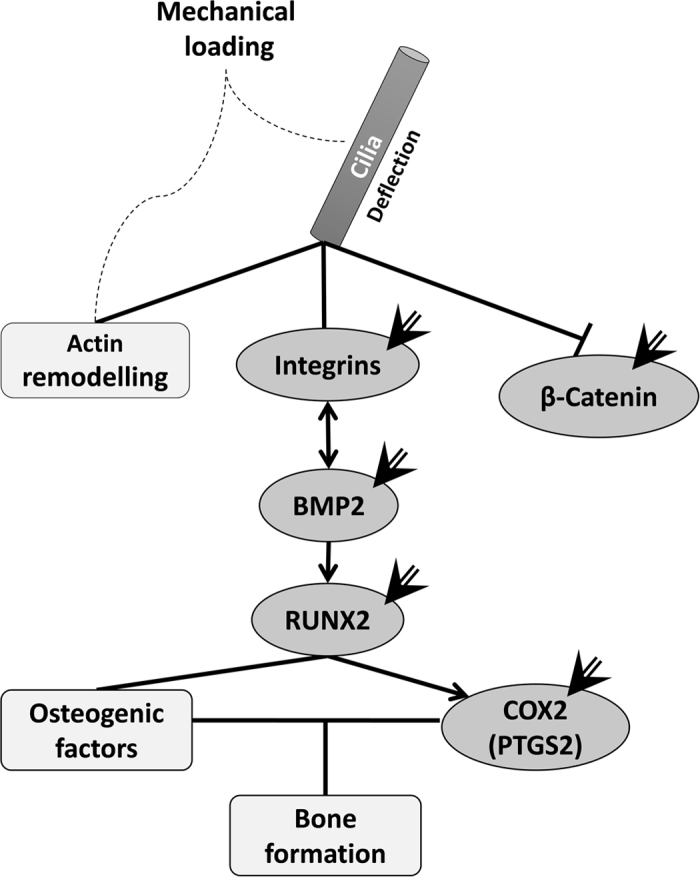
Illustrated molecules are connected through pathways linking ciliary mechanosensation to bone formation. The molecules that we show to be differentially affected in IS (marked by an arrow) are related through multiple interconnected pathways, summarized in this figure. The results of our gene expression studies are confirmed by expected responses through these pathways. For example, BMP2 expression directly affects RUNX2, which in turn affects COX2 expression.

Bones are under constant mechanical stimulation that is transferred to cells through cilia. Changes in mechanical loading have a regulatory effect on bone cell turnover through changes in gene expression^16^,^47^. We applied physiologically relevant mechanical stimulation to bone-derived cells and surveyed gene expression to investigate the effect of IS elongated cilia on cellular mechanotransduction. Bone morphogenic protein 2 (BMP2), Runt related transcription factor 2 (RUNX2) and Cyclooxygenase-2 (COX2) are involved in several interconnecting pathways that lead to osteoblastogenesis and bone formation^48^. Considering their established mechanosensitivity, the fact that all these genes showed a reduced response to mechano-stimulation in IS cells might explain why almost half (38% to 65% in different cohorts) of IS patients suffer from osteopenia and/or osteoporosis^49^,^50^. When we treated control cells with LiCl, we increased the length of their cilia and they also showed a dramatically decreased response for osteogenic factors upon mechanical stimulation (Fig. 4). Our results suggest that the lower bone mass density reported for IS patients could be the result of abnormalities in late stages of bone formation and mineralization, or perhaps as a consequence of their poor response to mechanical stimulation, which could be associated to the elongation of their cilia. COX2 (also known as Prostaglandin-Endoperoxide Synthase 2, PTGS2) is a cilia-dependent mechanosensitive gene in bone and the key enzyme in prostaglandin biosynthesis. Addition of prostaglandin has been associated with irregularity of cilia length in IMCD3 cells, and COX2 knockdown caused curved body axis in zebrafish (31%; n = 110)^51^. Interfering with beta 1-integrin signaling can reduce the normal upregulation of COX2 that occurs after mechanical stimulation in osteocytes^52^. Integrins are localized to the primary cilium^53^ and mechanical stimuli modulates integrin-mediated signals that are transmitted through focal adhesion sites and actin bundles^54^. In the case of the two integrins that we tested, beta 1 and beta 3, the response pattern to flow were found to be different between IS cells and controls. These trends further demonstrate the existence of an altered mechanosensation in IS cells, and reflect the *in silico* work of Khayyeri *et al*.^11^, where elongated cilia were modeled to show hypersensitivity to mechanical stimuli. Figure 5 illustrates how our tested genes are related through pathways that link ciliary mechanotransduction to bone formation. In response to mechanical stimulation, IS cells having elongated cilia showed an immediate elevation in CTNNB1 (beta-catenin) expression that increased throughout the 16-hour profile (Fig. 3), which was not seen in controls or LiCl treated cells. The mechanical response profiles for the other signaling molecules did not significantly differ among IS, control, or LiCl treated groups (Supplementary Fig. S3).

Bone mass homeostasis is the outcome of constant regulation of complex interactions between several systems including growth factors, hormones and mechanical loading. Wnt signaling has been shown to regulate bone remodeling, although different studies suggest different roles. *In vivo* administration of LiCl has been shown to increase bone mass in mouse models^55^ but not in supplemented chickens^56^. *In vivo*, inhibition of beta-catenin degradation in osteoblasts resulted in a high bone mass phenotype^57^. In addition, several studies suggest an inhibitory role for the Wnt pathway on osteoblastic differentiation^58^,^59^. Jansen *et al*.^60^ show that the role of Wnt in bone homeostasis is experimentally dependent on the type of cell examined, the stage of differentiation, interactions with other mechanosensitive pathways, and the duration of mechanical loading^60^. Our LiCl treated control cells showing a reduced biomechanical response for osteogenic factors similar to IS cells, could be the results of disturbed Wnt signaling which is required for later stages of osteoblast differentiation and matrix mineralization^58^.

Elongated cilia can be caused by decreased intracellular calcium, increased cyclic AMP, cyclic AMP-dependent PKA activity^61^, and modulation of the target-of-rapamycin (TOR) growth pathway^62^. Interestingly, increased cAMP levels were reported previously in IS osteoblasts and seems to be caused by a hypofunctionality of G inhibitory proteins^63^,^64^. The cilium is highly dynamic in its capacity to respond to extracellular stimulation and induce downstream intracellular changes. It is important to remember that we counted the number of ciliated cells and measured their lengths while they were in a stationary phase. We do not know how the cilia length changes in response to flow. Considering the dynamic nature of cilia, this could be interesting to study. Varying levels and duration of mechanical loads have an important role in cilia length regulation via an adaptive signaling mechanism^65^. Further insights could be gained from future studies investigating the IS cilia phenotype under different mechanical stimulations.

Ciliopathies comprise a large number of human genetic disorders that are defined by the causative or predisposing gene being related to cilia structure, function, sensory pathways, or localization. To examine whether the IS cilia phenotype is linked to ciliary genes, we reviewed established cilia gene lists for associations to spinal curvature and surveyed well defined IS genes in human and animal studies for a functional link to cilia. From our review using the SYSCILIA gold standard list of 303 verified ciliary genes, we found that 55 genes are associated with a human syndrome having clinical reports of scoliosis. Two of these genes, SUFU (Suppressor of fused homolog) and AJAP1 (Adherens junctions associated protein 1) are in loci that are associated with IS through linkage studies^66^,^67^, and 19 have both clinical (human) and experimental (animal model) associations with scoliosis. Furthermore, we found an additional 13 published animal model studies in which manipulation of the ciliary gene caused spinal curvature. In summary, 22% of these well-established cilia genes are associated with spinal curvature. In addition, from the study by Kim *et al*.^38^, we found 3 other genes that modulate ciliogenesis or cilia length and feature a clinical syndrome with reported scoliosis. Supplementary Table S2 (available) is a list of ciliary genes that are associated with a scoliosis phenotype.

All well supported IS genes, and several genetic animal models with a similar curve phenotype, are linked to cilia (Table 1). We tested two of the IS genes, Ladybird homeobox 1 (LBX1) and POC5, for differential mechanotransduction in our functional assay. We were unable to amplify LBX1 from our bone cells (data not shown), but POC5 showed a dramatic response after 4 hours of stimulation suggesting a role in mechanotransduction, although not exclusive to IS. POC5 in mammalian cells plays a role in stages coupled to cell cycle progression. POC5 is essential for centriole assembly and procentriole elongation. Procentriols can eventually mature to serve as the base of cilia^68^. Considering the initial depletion of POC5 expression in our results and the apparent recovery at later stages of flow application, it would be interesting to study the dynamics of cilia growth in response to shear stress in coordination with POC5 expression and localization.

Considering that many ciliary genes are associated with spinal curvature, and that IS genes are implicated in ciliary function or structure, we expected to find an enrichment of rare variants in ciliary genes in our French-Canadian cohort. From our exome analysis, the only ciliary gene among our candidates is FUZ, encoding a planar cell polarity protein that is involved in ciliogenesis and cell mobility. However, FUZ has a greater number of variants among controls, and we did not see a biomechanical response in its gene expression (among patients or controls). Among the 25 most significant genes in our list of candidates, we identified seven that have a greater number of variants among patients compared to controls. Interestingly, the majority of these genes are involved in elements of cellular mechanotransduction machinery. For example, Cytoplasmic linker associated protein 1 (CLASP1) not only interacts with actin filaments and regulates microtubules, but also is essential for maintaining spindle position and correct cell division axis^69^,^70^,^71^. CD1B and LYN also interact with actin^72^. LYN plays an important role in integrin signaling and regulates cell proliferation, migration, and differentiation^73^. SUGT1 and HNRNPD have roles in cell cycle and cell proliferation^74^,^75^. SUGT1 is also required for centrosome organization^74^. Our examination of variant profiles for patients used in our cellular analyses depict that all have variants in at least one of the following: CD1B, CLASP1, SUGT1 (Supplementary Fig. S4).

Although our reviews support a direct role for ciliary genes in IS, our exome data suggests that elongated cilia result from cellular processes affecting cilia indirectly. This is reinforced by our observations of elongated cilia prior to and during early cell cycle events. It is possible that idiopathic scoliosis is a result of heterogeneous gene defects pertaining to the cellular mechanotransduction machinery in general. Independent support for this idea comes from a recent IS exome study that reports rare variants in extracellular matrix (ECM) genes strongly contributing to disease risk^76^. Cilia extend outward from cells and protrude into the ECM environment to sense substrate changes. It has been shown that ECM molecules such as collagen are closely associated with primary cilia in chondrocytes, in which several well-known ECM receptors colocalize with primary cilia^43^,^53^.

This study identifies elongated cilia in idiopathic scoliosis patient bone cells as a new observation connected to the phenotype, and demonstrates functional consequences related to cellular mechano-responsiveness. Although validated IS genes are functionally or structurally linked to cilia, exome results suggest that the genetic architecture is more broadly related to biomechanics. Differential basal gene expression in IS cells compared to control cells treated with LiCl and untreated, suggest altered canonical Wnt signaling. Wnt signaling is known to affect bone homeostasis and biomechanical responsiveness^77^,^78^. We demonstrated an impaired biomechanical response in both IS cells and LiCl treated cells. However gene expression differences between the IS and LiCl induced elongated cilia phenotypes reflect a unique mechanism for the IS phenotype. Further studies are required to elucidate the signaling pathway(s) altered in IS and how these relate to genetic studies.

Considering the fact that both therapeutic options for IS, surgical (e.g. growth modulation with fusion-less devices as well as pedicle screw implantations) and nonsurgical (e.g. bracing) involve biomechanics, knowledge of the bodily response to mechanical stimuli is of crucial importance. Cilia have been shown to have a direct effect on bone formation in response to strains surrounding an intraosseous implant in mice^15^. Our work provides first evidences for involvement of primary cilia in idiopathic scoliosis and first indications towards a possible cellular-molecular mechanism underlying the disease^79^. An understanding of cellular defects affecting mechanotransduction in IS patient holds many novel possibilities for therapeutic innovations and a better understanding of phenotypic variability.

## Methods

### Study cohorts

This study was approved by the institutional review boards of The Sainte-Justine University Hospital, The Montreal Children’s Hospital, The Shriners Hospital for Children in Montreal and McGill University, as well as the Affluent and Montreal English School Boards. Parents or legal guardians of all the participants gave written informed consent, and minors gave their assent. All the experiments were performed in accordance with the approved guidelines and regulations. All the subjects are residents of Quebec and are of European descent. Each IS patient was clinically assessed by an orthopedic surgeon at the Sainte-Justine University Hospital. The inclusion criteria for this study was a minimum Cobb angle of 10 degrees and a diagnosis of idiopathic scoliosis. Cobb angle is the clinical parameter used for quantification of curve magnitude where a larger angle denotes a greater magnitude. For cellular experiments, we used bone samples from a subset of patients who required corrective surgery. Control bone samples were from surgical non-scoliotic trauma patients recruited at the Sainte-Justine University Hospital. All patients used for cellular studies were adolescent females (Table 4). The medical files of controls were reviewed to exclude the possibility of scoliosis. For exome sequencing, control blood samples were collected from non-IS participants that were first screened by an orthopedic surgeon using the forward-bending test (Adam’s test)^80^. Any children with apparent spinal curvature were not included in the control cohort.

### Cell culture

Primary osteoblasts were derived from bone specimens obtained from IS patients and trauma patients (as controls), intraoperatively. For all IS cases, bone specimens were surgically removed from affected vertebrae (the sampled vertebrae varied from T3 to L4) as a part of correctional surgery. For non-scoliotic control cases, bone specimens were obtained from other anatomic sites (tibia or femur) during trauma surgery. Using a cutter, bone fragments were manually reduced to small pieces under sterile conditions. The small bone pieces were incubated in αMEM medium containing 10% fetal bovine serum (FBS; certified FBS, Invitrogen Life Technologies, ON, Canada) and 1% penicillin/streptomycin (Invitrogen) at 37 °C in 5% CO_2_, in a 10-cm^2^ culture dish. After one month, osteoblasts emerging from the bone pieces were separated from the remaining bone fragments by trypsinization. Bone cells were characterized using alizarin red (Supplementary Fig. S5) and ALP staining (data not shown). In addition to the expression of osteoblast markers (RUNX2, SPP1 and BMP2) used in our qPCR experiment, we also confirmed the expression of Alkaline Phosphatase, Bone Sialoprotein II and Osteocalcein, using RT-PCR, as osteoblast specific genes in our primary cultures (Supplementary Fig. S5). For serum starvation and to promote ciliogenesis, cells were washed in PBS upon confluency and incubated in media supplemented with 1% FBS for the desired time periods (0 h, 24 h, 48 h, and 72 h). For shear stress experiments, cells were washed with PBS after starvation and incubated in regular media right before fluid flow application.

### Immunofluorescence

Cells were seeded in 8-well chamber slides (Falcon, Corning Incorporated, AZ, USA) at a density of 9 × 10^5^ cells per well. Upon reaching 80% confluence, the cells were washed with PBS and starved to induce cilia differentiation. At each time point during starvation, the cells were washed with PBS, fixed with 4% PFA in PBS buffer for 10 minutes at room temperature, washed (1% BSA in PBS), and then permeabilized with 0.1% Triton-X-100 in PBS for 10 min at room temperature. After two washes, the cells were blocked in 5% BSA in PBS for 1 h at room temperature. Mouse anti-acetylated α-tubulin (Invitrogen; 32–2700) diluted (1:1000) in 3% BSA-PBS was the primary antibody to detect cilia. Cells were incubated with this primary antibody overnight at 4 °C. The following day, after 3 washes, the cells were incubated for 1 h at room temperature with Alexa Fluor 488 conjugated goat anti-mouse secondary antibody (Invitrogen; A11029). After 3 washes, 1 μg/ml dilution of Hoechst (Sigma-Aldrich, ON, Canada; 94403) in 1% BSA-PBS was used to stain the nucleus at room temperature for 10 min. Alexa Fluor 555 Phalloidin dilution (1:40) in 1% BSA-PBS incubation at room temperature for 20 min was used to stain the cytoskeleton. The images were captured on a Leica Confocal TCS-SP8 or Zeiss Confocal 880 using × 63 (oil) objective with 1,024 × 1024 pixels resolution. Each sample has been examined in stitched 5 × 5 tile images, in duplicate (50 fields of view). Maximum projections of the Z-stacks were used for primary cilium measurement and counting was done in Image J (NIH). Two separate double staining with anti-Ninein antibody (Millipore, CA, USA, ABN1720) as the cilia base marker and anti-IFT88 (Proteintech, IL, USA, 13967-1-AP) were performed to co-stain the cilia alongside anti-acetylated α-tubulin to confirm the method.

### Proliferation assay

Bone cells acquired from IS patients were cultured, as previously described in the methods regarding cell culture. Upon reaching 90% confluence, cells were harvested by adding Trypsin-EDTA (0.25%) and phenol red (Thermo Fisher Scientific Inc., NY, USA 25200-072) for subculture (P3 to P5). Cells were washed and counted (Trypan Blue staining of viable cells) using the Vi-Cell XR (Beckman Coulter, Inc., CA, USA) automated cell counter and then seeded in 12 well plates (100,000 per well in triplicate for each sample). Cells were allowed to grow at 37 °C in 5% CO_2_ and each well was counted at different time points (24 h, 48 h, 72 h, 96 h and 120 h post culture). Bone cells from age- and gender-matched trauma surgical patients were used as controls.

### *In vitro* fluid flow stimulation

For each sample, the cells were divided equally between 4 vented 75 cm^2^ tissue culture flasks and cultured in 21 ml medium (αMEM + 10% FBS + 1% penicillin/streptomycin). Upon reaching 80% confluence, the medium was removed, the cells were washed with warm PBS and then transferred to starvation medium for 48 h. After 48 h cells were washed again and transferred back to 20 ml regular medium immediately before they were subjected to oscillatory fluid flow using a double-tier rocking platform, with some modification of the rocker method described by Robin M. Delaine Smith *et al*.^16^ with a maximum tilt angle of ±20 degrees, and a speed of 1 tilt per second (equal to 1 Hz). The entire unit was housed in a cell culture incubator held at 37 °C and 5% CO_2_ for the duration of the flow experiments (4 h, 8 h, and 16 h). No flow 0 h cells were housed in the same incubator and harvested at 8 h.

Fluid shear stress patterns were applied to cells in a predictable, controlled, and physiologically relevant manner through the whole experiment. From a biomechanical point of view, one expects that the cilia-related gene expression to be a function of time elapsed, t, and the shear stress exerted on the cells, which in turn depends on the fluid viscosity, *ν*, the frequency of flow oscillations, *f*, and the thickness of the fluid film, *h*. Designing an experiment in which all these parameters match the physiological conditions can be prohibitively challenging, and in fact unnecessary. Fortunately, the design of the experiment can be simplified using the 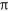-Buckingham theorem^81^ that is widely used in engineering and physics. The *π*-Buckingham theorem states that the dynamics of a problem (e.g. fluid flow) can be completely described and measured by a set of nondimensional quantities.

Here we define *ϕ* to be the ratio of gene expression measured at a given time, *t*, to its expression at 0 h. Noting that *ϕ* is a nondimensional quantity, one can use the *π* -Buckingham theorem to show that *ϕ* is a function of two other nondimensional numbers, equation (1):


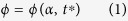


where *α* is the Womersley number defined as


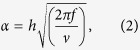


and t* is a nondimensionalized time


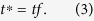


The Womersley number takes into account the effect of viscosity and shear stress exerted on the cell and is widely used in biomechanical studies involving pulsating fluid flow, equation (2)^82^. The value of Womersley number ranges from 5 to 18 in fluid motion of cerebrospinal fluid in the spinal cavity^26^. We designed our experiment such that the Womersley number experienced by the cells is equal to 8, which is well within the expected range *in vivo*. This value corresponds to an average shear stress at the center of the dish with a magnitude of 1 [Pa] in our experiment (see Zhou *et al*.^83^ for details of calculation).

### RNA extraction

All RNA was extracted using Trizol (Invitrogen-Thermo Fisher Scientific, 15596-026), according to the manufacturer’s instructions. Briefly, cell culture dishes containing adherent bone cells (passage 2 or 3) were washed with PBS before trypsinization, then transferred to a 15 ml tube and after centrifugation, the cell pellet was stored immediately at −80 °C. All the cells went through RNA extraction the following day and were lysed in 1 ml Trizol. RNeasy MinElute Cleanup Kit (Qiagen Inc., ON, Canada; 74204) was used to purifiy RNA, according to the manufacturer’s instructions.

### Quantitative RT-PCR

Reverse transcriptase quantitative PCR (RT-qPCR) was used to assay gene expression levels. All the primer design, validation, and gene expression was performed at the Genomics core facility of Institut de Recherche en Immunologie et Cancérologie (IRIC), University of Montreal, Quebec. All RNA was run on a bioanalyzer using a Nano RNA chip to verify its integrity. Total RNA was treated with DNase and reverse transcribed using the Maxima First Strand cDNA synthesis kit with ds DNase (Thermo Fisher Scientific). Before use, RT samples were diluted 1:5. Gene expression was determined using assays designed with the Universal Probe Library from Roche (www.universalprobelibrary.com). For each qPCR assay, a standard curve was performed to ensure that the efficacy of the assay is between 90% and 110%. Quantitative PCR (qPCR) reactions were performed in triplicate with 2 internal controls (GAPDH and HPRT) using PERFECTA qPCR FASTMIX II (Quanta Biosciences, Inc., MD, USA), 2 μM of each primer, and 1 μM of the corresponding UPL probe. The Viia7 qPCR instrument (Thermo Fisher Scientific) was used to detect the amplification level and was programmed with an initial step of 20 second at 95 °C, followed by 40 cycles of: 1 sec at 95 °C and 20 second at 60 °C. Relative expression (RQ = 2 − ∆∆CT) was calculated using the Expression Suite software (Thermo Fisher Scientific), and normalization was done using both GAPDH and HPRT. The baseline expression level at 0 h (before treatment) of every sample was defined as its own calibrator. The calibrator has a RQ value of 1 because it does not vary compared to itself. For each gene, the two groups (control and IS) were compared at each time point using a pairwise t-test. In this way, we asked one question per gene and we did three comparisons to answer (three comparisons per family of test). After looking at the results of these tests, we asked another question for three of the genes that seemed to show an overall expression profile that was different between IS and controls (ITGB1, CTNNB1 and POC5). For these genes we added three more comparisons: the expression at 0 h for IS was compared to each of the other time points (4 h, 8 h and 16 h) using 3 separate pairwise t-tests. We also examined gene expression values for IS, controls and LiCl treated controls at baseline, by using the delta Ct mean (expression level normalized with endogenous controls) of every sample at 0 h. The control cells were considered as the calibrator and the changes in gene expression of the other two groups were calculated as the fold changes in relative quantity (RQ) as explained above. A greater than ±2-fold gene expression change relative to control was considered significant.

RT–PCR primer sequences are as follows:

BMP2 F: 5′-cagaccaccggttggaga-3′; R: 3′-ccactcgtttctggtagttcttc-5′

SPP1 F: 5′-gcttggttgtcagcagca-3′; R: 3′-tgcaattctcatggtagtgagttt-5′

ITGB3 F: 5′-gggcagtgtcatgttggtag-3′; R: 3′-cagccccaaagagggataat-5′

PTGS2 F: 5′-gctttatgctgaagccctatga-3′; R: 3′-tccaactctgcagacatttcc-5′

RUNX2 F: 5′-ggttaatctccgcaggtcac-3′; R: 3′-ctgcttgcagccttaaatga-5′

ITGB1 F: 5′-cgatgccatcatgcaagt-3′; R: 3′-acaccagcagccgtgtaac-5′

POC5 F: 5′-aacaactgtgtaatcagatcaatgaa-3′; R: 3′-tgcctatggcatgagacaag-5′

LBX1 F: 5′-tcgccagcaagacgttta-3′; R: 3′-gccgcttcttaggggtct-5′

FUZ F: 5′-tcacctccacgcacttcc-3′; R: 3′-gggcctggtagacctcatct-5′

GAPDH F: 5′-agccacatcgctcagacac-3′; R: 3′-gcccaatacgaccaaatcc-5′

HPRT F: 5′-tgatagatccattcctatgactgtaga-3′; R: 3′-caagacattctttccagttaaagttg-5′

AXIN2 F: 5′- ccacccttctccaatcc-3′; R: 3′-tgccagtttctttggctctt-5′

INVS F: 5′-tgataacttatttcgaaccccact-3′; R: 3′-acaatctgtgcatggcctaa-5′

GSK3B: 5′-ttggagccactgattatacctct-3′; R: 3′-tcccctggaaatattggttg-5′

PTCH1: 5′-catcaactggaacgaggaca-3′; R: 3′-gcgacactctgatgaaccac-5′

GLI1: 5′-tgaaactgactgccgttgg-3′; R: 3′-ggatgtgctcgctgttgat-5′

### Induced phenotype via Lithium Chloride (LiCl)

To examine the effect of LiCl on cilia length of primary osteoblasts, we exposed the previously tested controls to 1 mM, 10 mM and 50 mM LiCl (Sigma-Aldrich, ON, Canada;203637). Osteoblast were starved for 48 hours and treated for 24 hours before measuring the length of their cilia (Supplementary Fig. S2). Cells that were treated with 50 mM LiCL did not survive. Cells undergoing mechanical stimulation were treated with 10 mM LiCl.

### Exome and Sanger sequencing

Genomic DNA was extracted from the whole blood of 73 IS patients and 70 controls using the PureLink Genomic DNA extraction kit (Thermo Fisher Scientific). Library preparation and exome sequencing was performed at GENESE (Génomique de la Santé de l’Enfant, Sainte-Justine University Hospital Research Center). Selected variants were confirmed using Sanger sequencing technologies at the Genome Quebec Innovation Centre. Samples were barcoded, and captured using libraries of synthetic biotinylated RNA oligonucleotides (baits) targeting 50 Mb of genome (Agilent SureSelect Human All Exon 50 Mb v3), and sequenced on the 5500 SOLiD Sequencing System (Thermo Fisher Scientific). Trimmed FASTQ formatted sequences were aligned to the exome target sequence using Bfast +bwa (version 0.7.0a) in the paired-end alignment mode^84^. Mapped reads were refined using GATK and Picard program suites^35^ to improve mapped reads near indels (GATK indel realigner) and improve quality scores (GATK base recalibration) and to remove duplicate reads with the same paired start sites (Picard mark Duplicates). Variants were called using SAMTOOLS batch calling procedure referenced against the UCSC assembly hg19 (NCBI build 37). Variants were additionally filtered to remove variants that are present with minor allele frequencies (MAF) >0.05 (dbSNP, 1000 genomes, ExAC and/or Exome variant server (ESP). Variants were annotated using the GEMINI framework^85^ that provides quality metrics and extensive metadata (e.g. OMIM, clinVar, etc.) to help further prioritize variants. To optimize the querying criteria for the GEMINI database, we performed bidirectional Sanger sequencing for more than 100 different variants. Using an optimized threshold (Coverage DP > 10x, Genotype quality GQ > 80, Call rate >90%, Alternate quality (QUAL) >50, Map quality >20), the results show 85% genotype correlation between the sequencing methods. This threshold was used to filter our data prior to analysis.

### Statistical analyses

To test for accumulation of rare variants in genes associated with IS, we used the Sequence Kernel Association Optimal unified test algorithm SKAT-O^36^. SKAT-O is a region-based omnibus test that increases a study’s power to detect rare variants. Because there is no model for the genetic basis underlying IS, SKAT-O is optimal over SKAT or burden testing alone, since it is a robust technique to detect variable effect rare polymorphisms^22^. Variants that passed our filtering criteria, with a dataset minor allele frequency ≤5% were analyzed in two different sets (a complete list of variants is presented in Supplementary Table S3). Additionally, high quality variants with membership to the Illumina HumanExome Chip were extracted from the Gemini database for population structure analyses using R package SNPRelate. The top two components were used as covariates in the SKAT-O analysis. The first set used the manual recommended settings for rare variants: SKATBinary with snp weighting based on Madsen and Browning weights (i.e. less frequent are more impactful) (B1 = 0.5, B2 = 0.5). The second set weighted-snps are based on Combined Annotation Dependent Depletion (CADD) scores (i.e. functional, deleterious, and disease-causing variants have greater impact)^86^. For both sets, we generated a null model of no association between genetic variables and outcome phenotype adjusting for covariates. Covariate analysis confirmed that there was not population stratification in our dataset. The gene-level significant thresholds were determined by the efficient resampling (ER) method and the conservative minor allele count (MAC) threshold of ≤40^87^. To examine the number of ciliary genes in our datasets, we used two reference lists that define a ciliary function based on experimental evidence: (1) the SYSCILIA gold standard list of genes containing 303 ciliary genes verified by independent publications^37^; (2) a list of 52 genes (51 novel genes because one is in the SYSCILIA list) from a functional genomic screen that used RNA interference to identify genes involved in the regulation of ciliogenesis and cilia length^38^.

### Review of ciliary genes associated with spinal curvature

If idiopathic scoliosis is a genetically heterogeneous ciliopathy-like condition, then we expect a large number of known ciliopathies have spinal curvature as a comorbidity. We reviewed the SYSCILIA gold standard gene list and the Kim *et al*. list^37^,^38^ to ascertain how many ciliary genes were associated with spinal curvature phenotypes in either a human syndrome or an animal model. For each of the 303 genes, the search terms in Google included the gene name with “spinal curvature” and “scoliosis”. Additionally, if the gene was known to be associated with a syndrome in the OMIM database, we also searched the syndrome name with “scoliosis”.

## Additional Information

**How to cite this article:** Oliazadeh, N. *et al*. Identification of Elongated Primary Cilia with Impaired Mechanotransduction in Idiopathic Scoliosis Patients. *Sci. Rep.* **7**, 44260; doi: 10.1038/srep44260 (2017).

**Publisher's note:** Springer Nature remains neutral with regard to jurisdictional claims in published maps and institutional affiliations.

## Supplementary Material

Supplementary Figures

Supplementary Table S1

Supplementary Table S2

Supplementary Table S3

## Figures and Tables

**Table 1:** IS-associated genes in humans and/or animal models, which are also associated with cilia.

Gene	Scoliosis Association	Cilia Association
TBX6	PMIDs: 26120555, 20228709 (Congenital and idiopathic scoliosis in humans)	PMIDs: 18575602 & 17765888 (Affects morphology and motility of nodal cilia in mice & zebrafish)
LBX1	PMIDs: 26394188, 25987191, 25675428, 24721834 (Idiopathic scoliosis association in several ethnic groups, confirmed using different approaches)	PMIDs: 18541024 (deleted in a mouse model of the primary ciliary dyskinesia gene)
GPR126	PMIDs: 25954032, 25479386, 23666238 (Idiopathic scoliosis in humans and mice)	PMIDs: 16875686, 24227709 (No direct relation to cilia. Essential for the development of myelinated axons in zebrafish and mice)
PAX1	PMIDs: 25784220, 19080705, 16093716 (Congenital and idiopathic scoliosis in humans and mice)	PMIDs: 19517571, 23907320, 24740182 (Other family members are associated with cilia signaling pathways or ciliated tissues)
POC5	PMID: 25642776 (Idiopathic scoliosis in humans)	PMID: 23844208, 19349582 (interacts with cilia and is essential for centriole structure in humans and Drosophila)
KIF6	PMID: 25283277 (idiopathic-type curvature in zebrafish)	PMID: 16084724 (Predicted to be involved in ciliary function or structure)
PTK7	PMID: 25182715 (idiopathic-type curvature in zebrafish)	PMID: 20305649 (Role in cilia orientation in zebrafish)
FGF3	PMID: 25852647, 24864036 (Idiopathic scoliosis in a KO mouse model; Scoliosis in a human case report carrying loss-of-function mutation in the gene)	PMID: 26091072 (Affecting the organization of chondrocyte primary cilia in the growth plate in mice)

**Table 2:** Significantly longer cilia in IS-derived bone cells.

	0 h	24 h	48 h	72 h
Controls	1.94 ± 0.35	1.99 ± 0.32	2.05 ± 0.66	2.16 ± 0.78
IS	2.66 ± 1.14	2.87 ± 1.84	2.82 ± 0.81	2.80 ± 0.65
P Value	2.62632 E-22	1.00327 E-20	2.49 E-25	1.03284 E-14

The average length of cilia in μm ± variance at four starvation time points (0, 24 h, 48 h, 72 h). To compare the length of cilia between IS and non-IS controls, we combined measurements of up to 1000 cilia for each sample (25 fields per sample, in duplicate), at each time point, then used a t-test to compare the mean lengths of cilia in IS vs. control pools. The difference in length was significant across all the time points for IS patients (P value ≤ 0.005), n = 8 (4 IS vs. 4 Controls).

**Table 3:** The basal level of gene expression in LiCl-treated control and IS compared to non-treated control osteoblasts.

	RQ (Fold change)		
	Control	LiCl-Control	IS
BMP2	1	1.045	0.754
PTGS2	1	3.942*	1.481
RUNX2	1	0.202*	0.299*
SPP1	1	0.366*	2.020*
CTNNB1	1	3.367*	5.762*
GSK3B	1	0.296*	1.007
INVS	1	0.746	1.127
AXIN2	1	0.821	0.508
PTCH1	1	0.721	0.528
GLI1	1	Not Determined	0.687
ITGB1	1	1.670	1.169
ITGB3	1	0.692	0.829
POC5	1	0.499	1.133

The expression of several signaling and osteogenic factors were compared in human primary osteoblast samples from controls, LiCl treated controls and IS groups. The control cells are considered as the calibrator and the changes in gene expression of the other two groups are shown as the fold changes in relative quantity (RQ). LiCl-Controls were treated with 10 mM LiCl for 24 h. 2-fold change in gene expression compared to controls is considered significant and marked with an asterisks.

**Table 4:** Clinical features of patients tested for ciliary morphology.

Patient ID	Cobb Angle (degree)	Curve Type
1	42°–66°–38°	lTrTlL
2	21°–50°–67°–31°	lTrTlTLrL
3	50°–56°	lTrL
4	50°–89°	rTlTL

All samples (patients and controls) are from female subjects. Mean age for controls is 15 ± 3, mean age for patients is 15 ± 1. Cobb angles in degrees, multiple angles reflect multiple curves. Curve types - T: Thoracic; L: Lumbar; TL: Thoracolumbar; l: left and r: right.
