# Supplementary material for: Identification of Elongated Primary Cilia with Impaired Mechanotransduction in Idiopathic Scoliosis Patients
Source: Sci Rep. 2017 Mar 14;7:44260. doi: 10.1038/srep44260 (PMC5349607; doi:10.1038/srep44260)
Supplement: Supplementary Figures [file srep44260-s1.pdf]

Identification of Elongated Primary Cilia with Impaired Mechanotransduction in Idiopathic Scoliosis Patients

Niaz Oliazadeh[1, 2], Kristen Fay Gorman[1, 2], Robert Eveleigh[3], Guillaume Bourque[4], Alain Moreau[1, 2, 5] *

[1] Viscogliosi Laboratory in Molecular Genetics of Musculoskeletal Diseases, Sainte-Justine University Hospital Research Center, Montreal, Quebec, Canada, H3T 1C5

[2] Department of Biochemistry and Molecular Medicine, Faculty of Medicine, Université de Montreal, Montreal, Quebec, Canada, H3T 1J4

[3] Genome Quebec Innovation Center, McGill University, Montréal, Quebec, Canada, H3A 0G1

[4] McGill University, Montréal, Quebec, Canada, H3A 1A4

[5] Department of Stomatology, Faculty of Dentistry, Université de Montréal, Montreal, Quebec, Canada, H3A 1J4

* Contact information: Alain Moreau, PhD, Sainte-Justine University Hospital Research Center, Viscogliosi Laboratory in Molecular Genetics of Musculoskeletal Diseases (room 2.17.027), 3175 Côte-Sainte Catherine Road, Montreal, Quebec, H3T 1C5. Phone : 514-345-4931 ext. :5722. Fax : 514-345-4801. Email : alain.moreau@recherche-ste-justine.qc.ca

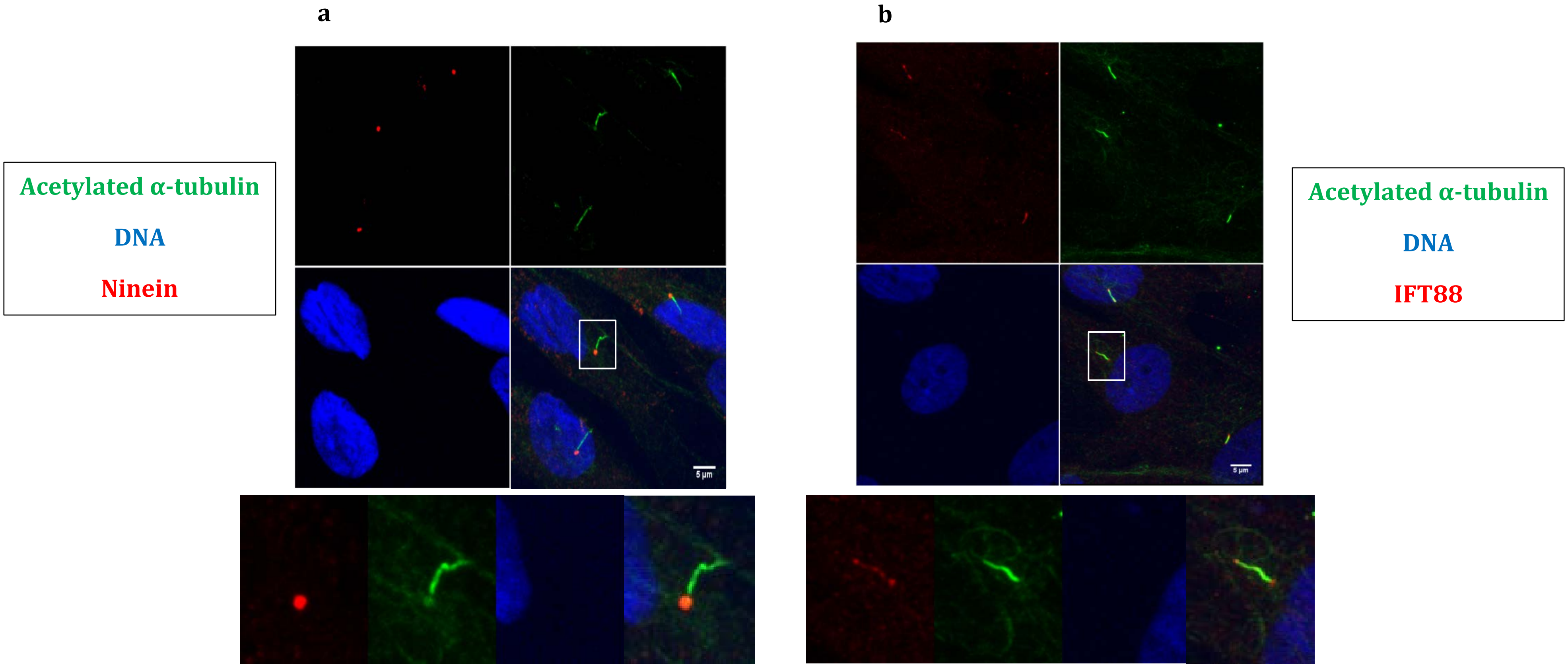

Supplementary Figure S1. Cilia Immunofluorescence co-staining. To validate the staining of cilia, double immunostaining was performed on fixed osteoblasts using Anti-acetylated α-Tubulin and **a)** anti-Ninein, as the basal body marker or **b)** anti-IFT88 to stain the length of cilia. Lower parts of each panel show the magnified version of the area framed in white rectangles from the upper part.

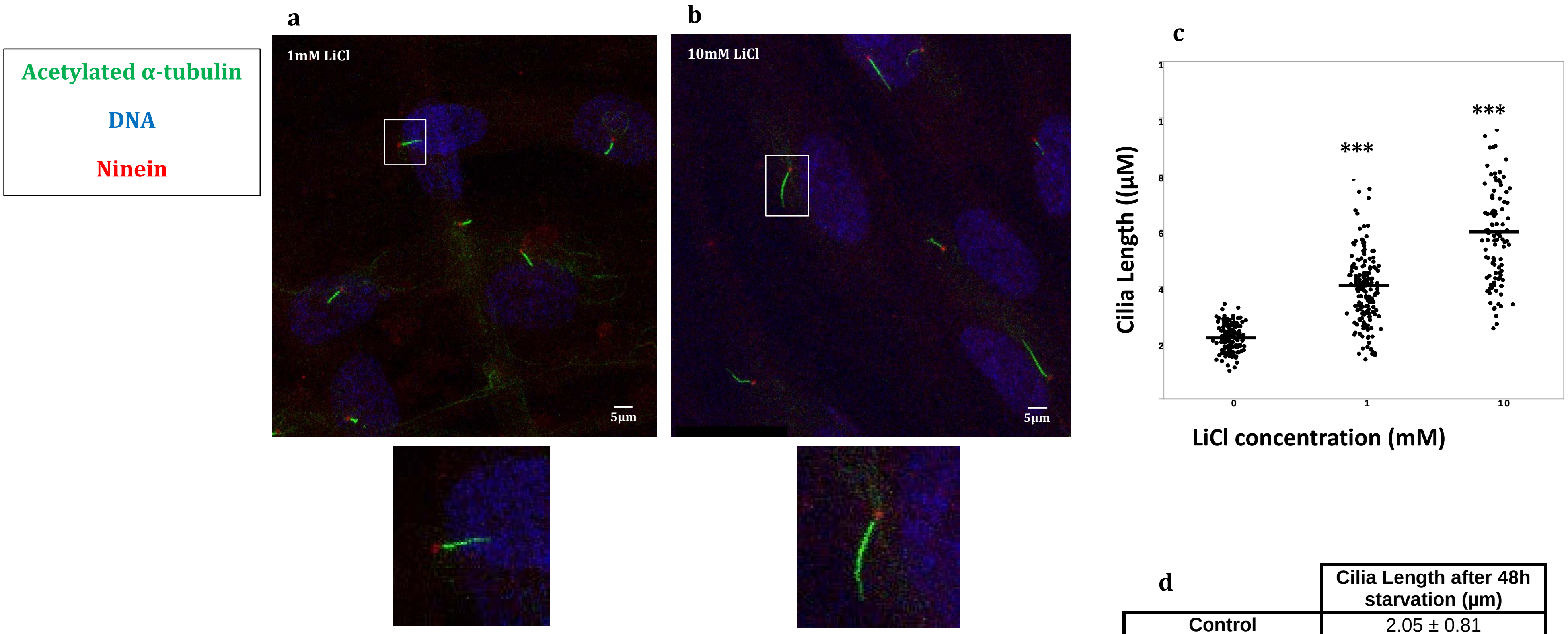

d

	Cilia Length after 48h starvation (µm)
**Control**	2.05 ± 0.81
**Control-1mM LiCl**	3.94 ± 1.24
**Control-10mM LiCl**	5.81 ± 1.72
**IS**	2.82 ± 0.90

Supplementary Figure S2. LiCl increases the length of cilium in primary human osteoblasts. Human primary osteoblasts (OB) were transferred to differentiation media (1% supplemented media) upon confluency, after 24h media was changed to differentiation media plus indicated concentrations of LiCl (1, 10 and 50 mM). The increased length of cilia is visible in immunofluorescence stained cilia of cells after **a)** 1mM LiCl and **b)** 10 mM LiCl. **c)** Both concentrations of LiCl significantly increase the length of cilia ($p < 0.0001$). The horizontal bars indicate the average value for each sample. Samples assayed in duplicate in 3 x 3 stitched images (18 fields) per sample. Statistical analysis was performed with Anova, followed by Tukey test using JMP-12®. **d)** The average length of cilia in µm ± variance.

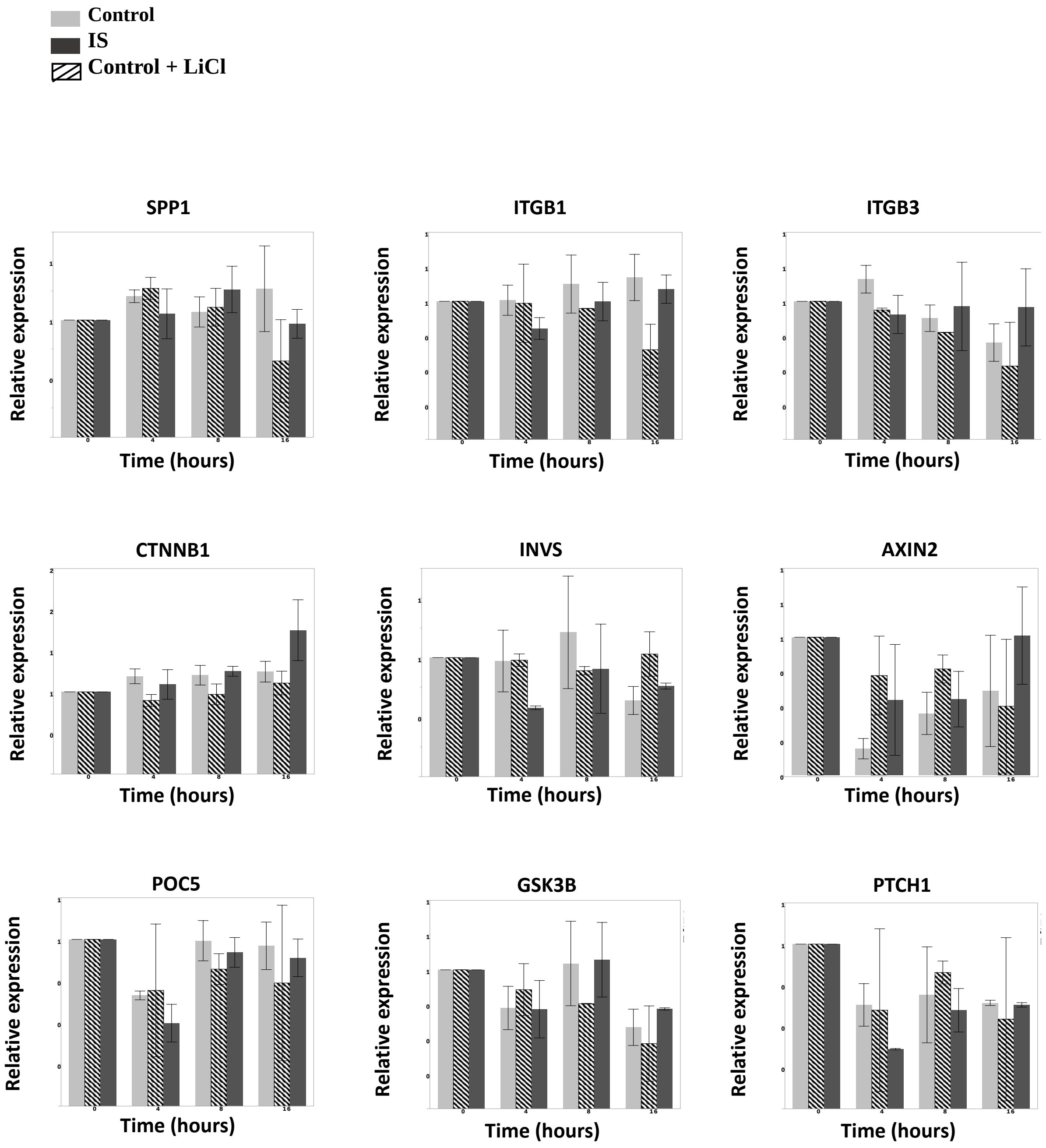

Supplementary Figure S3. Effect of 10 mM LiCl treatment on biomechanical response profile of control cells. Changes in gene expression following 10mM LiCl treatment for 24h in human primary osteoblasts in controls compared to not treated controls and IS cells is shown after 4, 8 and 16 hours of fluid flow mechanical stimulation. The 0h of every sample has been defined as its calibrator. The graphs represent the fold changes at each time point, compared to the calibrator. 10 mM LiCl significantly suppress the expression of BMP2 and PTGS2 ($p < 0.0001$). Statistical analysis was performed with Anova, followed by Tukey test using JMP-12®. Further details are exactly similar to Fig. 3 in the body of the paper.

ID #	ATP5B	BTN1A1	CD1B	CDK11A	CLASP1	DDX5	FBXL2	HIVEP1	HSD17B14	KCNMA1	PXDN	RAB31	RBM5	RNF149	SOD2	SUGT1	TOPBP1	ZCCHC14	ZNF323
1	0	0	2	0	0	0	0	0	1	0	1	2	0	1	0	1	1	2	0
2	1	1	1	2	2	1	0	0	0	1	0	0	1	1	2	1	1	0	0
3	1	0	0	3	1	1	0	0	1	1	1	0	0	0	0	0	0	0	1
4	0	1	1	3	1	0	2	4	0	0	0	0	1	1	0	0	0	3	1

Supplementary Figure S4. Mutation profile of the tested IS patients. Patients used in our cellular assays were surveyed for variants in genes listed in Supplementary Table 1 (significant genes from our SKAT-O analyses). Patients are listed as rows and each column is a gene. This heat map illustration shows a color coded profile of the number of variants per patient for a given gene. Only genes with a total of more than 1 variant are listed.

a

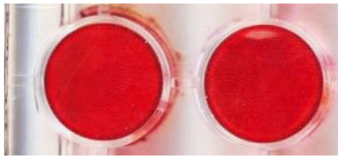

b

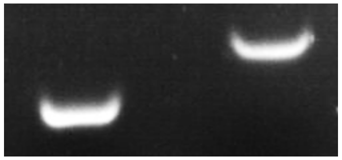

ALP BSP

c

**ALP**	Primer-F	CTGCAAGAAAGGGGACCCAAGAAA
	Primer-R	TGGGAGGGTCAGATCCAGAATGTT
**BSP**	Primer-F	TGTCACTGGAGCCAATGCAGAA
	Primer-R	CGAAAATGGTAGCCGGATGCAAAG

Supplementary Figure S5. Charachterization of osteoblast cells. We derived osteoblasts from surgical bone specimens. To confirm that these cells are osteoblasts, **a)** we show the results of Alazarin red staining. Mineralization was induced on confluent mono layer by addition of Ascorbic acis (50 µg/ml), Beta-glycerophosphate (2.5 mM) and Dexamethasone (10 nM). After 4 weeks of treatment cell were fixed with formaldehyde and stained with Alizarin red. Also, in addition to the RT-qPCR performed in this study using osteoblast genes (RUNX2 and SPP1), **b)** We performed RT-PCR on Alkalin phpsphatase (ALP) and Bone Sialoprotein II (BSP) to demonstrate the expression of bone markers in our cultured cells. **c)** Shows the sequence of the primers used for RT-PCR.
